# Meta-analysis of brain iron levels of Parkinson’s disease patients determined by postmortem and MRI measurements

**DOI:** 10.1038/srep36669

**Published:** 2016-11-09

**Authors:** Jian-Yong Wang, Qing-Qing Zhuang, Lan-Bing Zhu, Hui Zhu, Ting Li, Rui Li, Song-Fang Chen, Chen-Ping Huang, Xiong Zhang, Jian-Hong Zhu

**Affiliations:** 1 Department of Neurology, the Second Affiliated Hospital, Wenzhou Medical University, Wenzhou, Zhejiang 325000, China; 2 Department of Preventive Medicine, Wenzhou Medical University, Wenzhou, Zhejiang 325035, China; 3 Key Laboratory of Watershed Science and Health of Zhejiang Province, Wenzhou Medical University, Wenzhou, Zhejiang 325035, China

## Abstract

Brain iron levels in patients of Parkinson’s disease (PD) are usually measured in postmortem samples or by MRI imaging including R2* and SWI. In this study we performed a meta-analysis to understand PD-associated iron changes in various brain regions, and to evaluate the accuracy of MRI detections comparing with postmortem results. Databases including Medline, Web of Science, CENTRAL and Embase were searched up to 19^th^ November 2015. Ten brain regions were identified for analysis based on data extracted from thirty-three-articles. An increase in iron levels in substantia nigra of PD patients by postmortem, R2* or SWI measurements was observed. The postmortem and SWI measurements also suggested significant iron accumulation in putamen. Increased iron deposition was found in red nucleus as determined by both R2* and SWI, whereas no data were available in postmortem samples. Based on SWI, iron levels were increased significantly in the nucleus caudatus and globus pallidus. Of note, the analysis might be biased towards advanced disease and that the precise stage at which regions become involved could not be ascertained. Our analysis provides an overview of iron deposition in multiple brain regions of PD patients, and a comparison of outcomes from different methods detecting levels of iron.

Iron overload has been implicated in the pathology and pathogenesis of Parkinson’s disease (PD). The substantia nigra, where the selective loss of dopaminergic neurons occurs, is the primary region in the brain known to deposit iron. Additionally, aberrant iron concentrations have been observed in other brain regions such as red nuclei, globus pallidus and cortex of PD patients, despite of unknown pathology^1^,^2^,^3^. Spectroscopic analyses of postmortem brains display an increased iron levels in the substantia nigra, which has been suggested to correlate with the severity of PD^2^,^4^. In recent decades, advancements in imaging techniques, such as magnetic resonance imaging (MRI), have contributed to an enhanced understanding of the pathological progression and clinical diagnosis of PD. Consequently, iron load may be estimated in a non-invasive manner using R2/R2* relaxometry (with better results obtained using R2* 5,6,7) and, more recently, susceptibility-weighted imaging (SWI). Nonetheless, while largely consistent and reproducible results can be obtained in many experiments these techniques are not yet fully validated^8^.

In this study, we extracted results of iron analyses employing postmortem brains and R2* and SWI methods from the literature, and performed a systematical meta-analysis aiming to 1) confirm the iron overload observation in the substantia nigra, 2) explore other regions of the brain carrying different levels of iron, and 3) evaluate to what extent these two MRI methods correlate with the measurements of postmortem brains. Meanwhile, as detailed in the discussion section, several limitations are disclosed in an attempt to fully understand the scope of this meta-analysis, such that the disease severity was not differentiated due to insufficient information during data extraction that may affect outcomes of MRI imaging.

## Results

### Search Results

**Figure 1:**
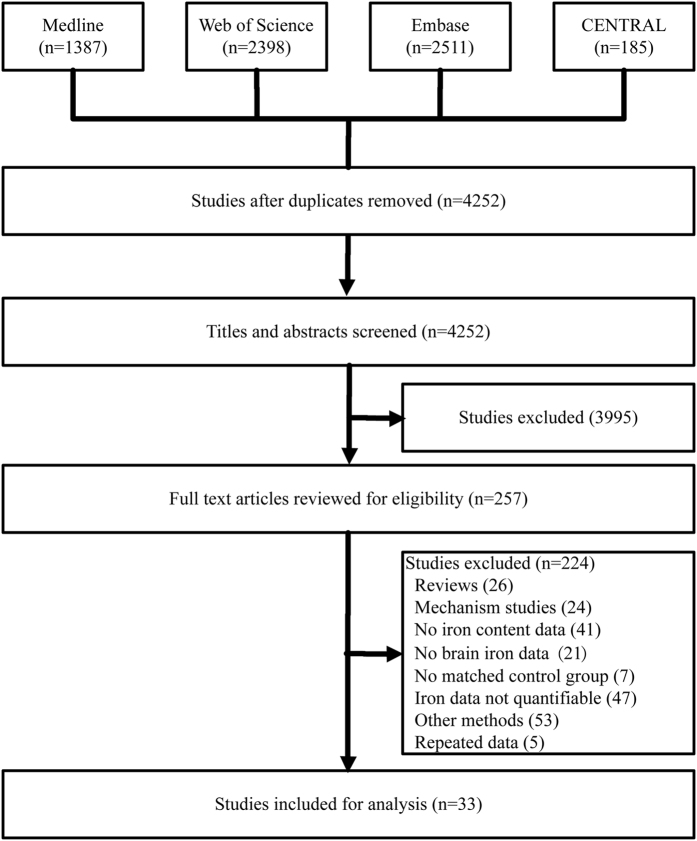
Flow chart describing the selection process of articles retrieved from initial literature search. CENTRAL, Cochrane Central Register of Controlled Trials.

The initial search using the keywords as described in the method section returned a total of 4252 articles (Fig. 1). A subsequent screening of the titles and abstracts reduced the number to 257. Following an exhaustive examination of the contents, 224 articles were excluded according to the selection criteria detailed in the method section. Of the 33 articles being selected that report iron content (summarized in Table 1), 11 of them employed postmortem analyses^2^,^4^,^9^,^10^,^11^,^12^,^13^,^14^,^15^,^16^,^17^, 14 were measured by R2* 3,18,19,20,21,22,23,24,25,26,27,28,29,30 and 8 by MRI relaxometry SWI^31^,^32^,^33^,^34^,^35^,^36^,^37^,^38^. The disease comorbidity and diagnostic performance of the cohorts of these 33 studies are summarized in Table S1.

### Quality Assessment

Quality assessment by Newcastle-Ottawa Scale suggested four-stars or above out of a maximum of nine for all of the 33 publications. The detailed quality assessment is listed in Table 1.

### Postmortem comparison of iron concentration in defined brain regions

**Figure 2:**
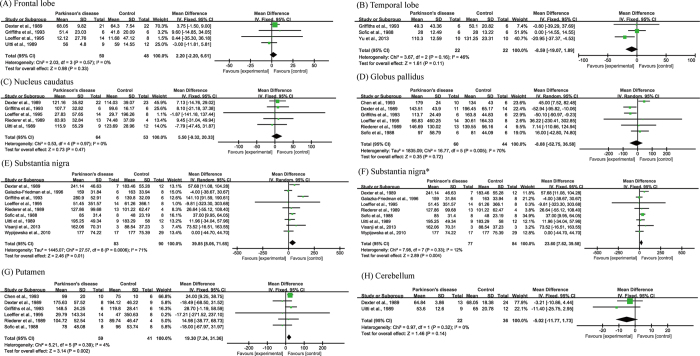
Statistical summaries and forest plots of studies comparing iron concentrations by postmortem analysis. (**D**,**E**) Pooled using random-effects models. The others were pooled using fixed-effects models. *Analyzed after heterogeneity was removed.

**Figure 3:**
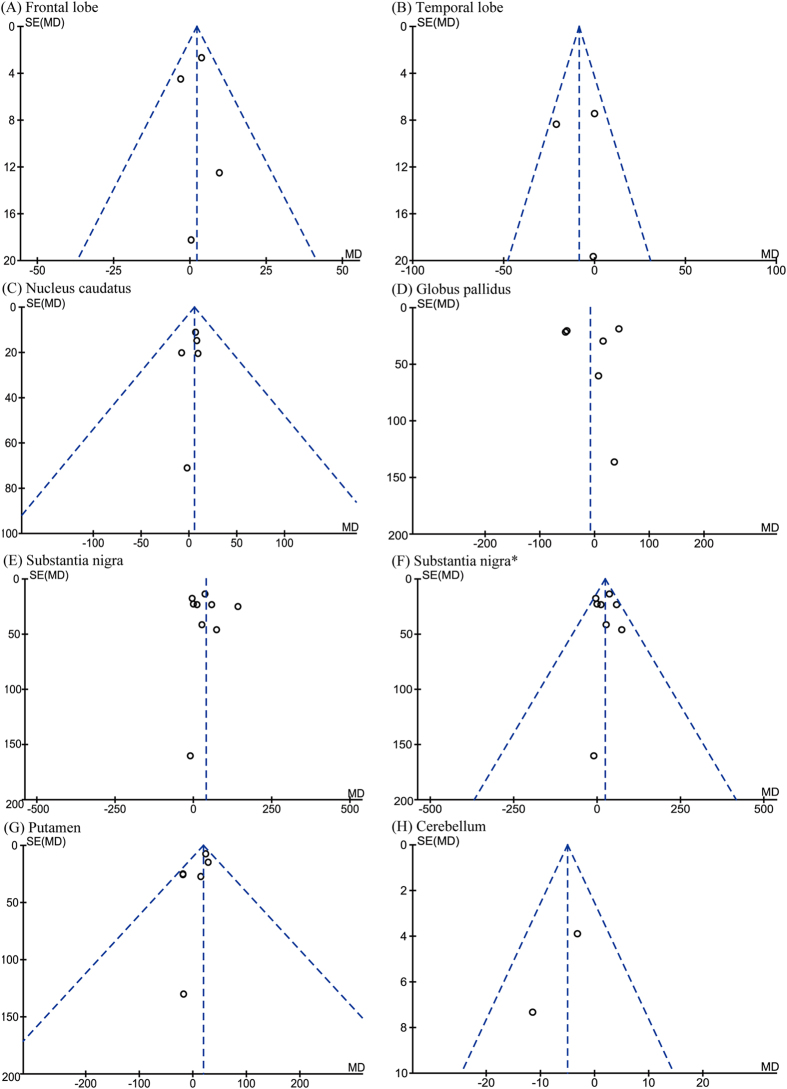
Funnel plots that examine possible publication bias in the studies by postmortem analysis. *Analyzed after heterogeneity was removed.

Eleven of the manuscripts examined iron concentration in seven regions of postmortem brains. The numbers of subjects for each region were 98 (frontal lobe), 44 (temporal lobe), 117 (nucleus caudatus), 104 (globus pallidus), 173 (substantia nigra), 100 (putamen), and 58 (cerebellum). Although iron concentration was significantly increased in the substantia nigra of PD patients (WMD = 39.85, 95% CI, 8.06–71.65, *p* = 0.01; Fig. 2E), significant heterogeneity was detected in these cohorts (I^2^ = 71%; *p* = 0.0006). Subsequent sensitivity analysis suggested that such heterogeneity was attributed to the study of Griffiths *et al*.^11^. Further analysis that eliminated this study (I^2^ = 12%; *p* = 0.33) also showed a significant increase of iron concentration in the substantia nigra (WMD = 23.60, 95% CI = 7.62–39.58, *p* = 0.004; Fig. 2F). Additionally, increased iron levels were observed in the putamen of PD subjects (WMD = 19.30, 95% CI  = 7.24–31.36, *p* = 0.002, I^2^ = 4%; Fig. 2G). No significant differences were observed in other brain regions (Fig. 2). The funnel plots analyzing publication bias appeared to be symmetric by visual inspection (Fig. 3).

### MRI comparison of iron concentration in defined brain regions

**Figure 4:**
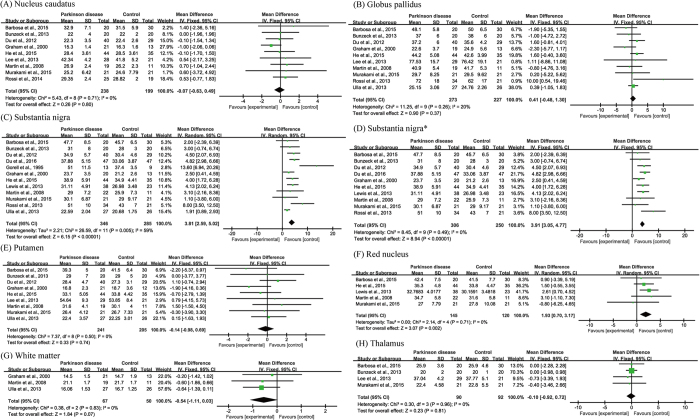
Statistical summaries and forest plots of studies comparing iron concentrations by MRI R2* relaxometry. (**C**) Pooled using random-effects models. The others were pooled using fixed-effects models. *Analyzed after heterogeneity was removed.

**Figure 5:**
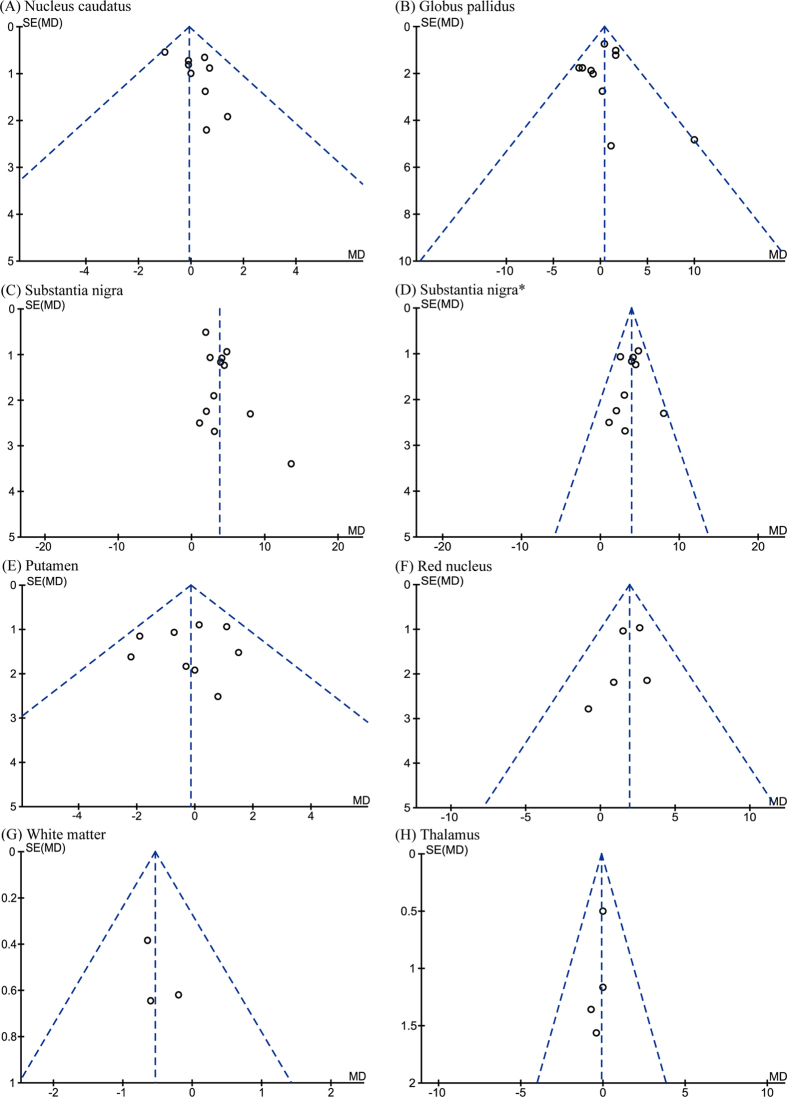
Funnel plots that examine possible publication bias in the studies by R2*. *Analyzed after heterogeneity was removed.

Fourteen articles were included in the R2* subgroup of meta-analyses in seven brain regions. The total subject numbers were 437 (nucleus candatus), 500 (globus pallidus), 631 (substantia nigra), 446 (putamen), 265 (red nucleus), 117 (white matter) and 182 (thalamus). In the substantia nigra of PD subjects, iron content was elevated (WMD = 3.81, 95% CI = 2.59–5.02, *p* < 0.00001) despite of a relatively high heterogeneity (I^2^ = 59%, *p* = 0.005; Fig. 4C). Results of a sensitivity analysis ascribed the heterogeneity to the studies of Ulla *et al*.^25^ and Gorell *et al*.^18^, as exclusion of them eliminated the heterogeneity (I^2^ = 0%, *p* = 0.49 ; Fig. 4D). Subsequent meta-analysis again demonstrated a significant increase of iron concentration in the substantia nigra (WMD = 3.91, 95% CI = 3.05–4.77, *p* < 0.00001; Fig. 4D). Iron concentration was significantly increased in the red nucleus (WMD = 1.93, 95% CI = 0.70–3.17, *p* = 0.002, I^2^ = 0%; Fig. 4F), but not in other brain regions (Fig. 4). The publication biases were acceptable as determined by funnel plots (Fig. 5).

**Figure 6:**
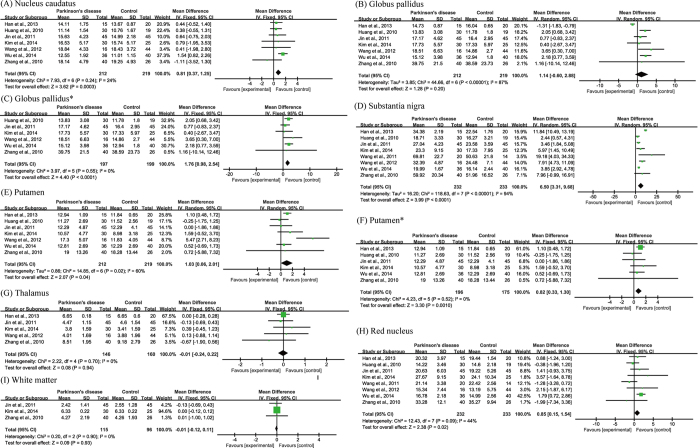
Statistical summaries and forest plots of studies comparing iron concentrations by SWI relaxometry. (**B**,**D**,**E**) Pooled using random-effects models. The others were pooled using fixed-effects models. *Analyzed after heterogeneity was removed.

**Figure 7:**
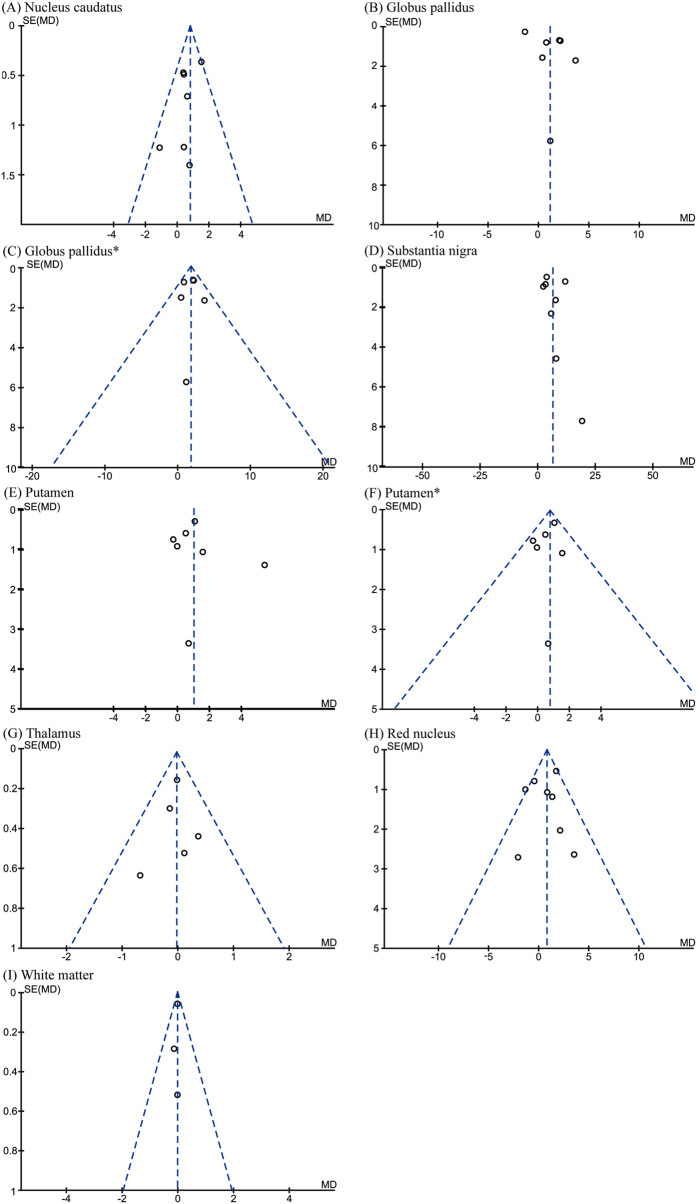
Funnel plots that examine possible publication bias in the studies by SWI. *Analyzed after heterogeneity was removed.

Eight articles were included in the SWI subgroup of meta-analyses in seven brain regions. The total subject numbers were 431 (nucleus caudatus), 431 (globus pallidus), 431 (putamen), 306 (thalamus), 465 (substantia nigra), 465 (red nucleus) and 211 (white matter). A significant increase in iron concentration was observed in the substantia nigra (WMD = 6.5, 95% CI = 3.31–9.68, *p* < 0.0001) with high heterogeneity (I^2^ = 94%, *p* < 0.0001; Fig. 6D). Significant increases in iron concentration were also shown in the nucleus caudatus (WMD = 0.81, 95% CI = 0.37–1.25, *p* = 0.0003, I^2^ = 24%; Fig. 6A), putamen (WMD = 1.03, 95% CI = 0.06–2.01, *p* = 0.04, I^2^ = 60%; Fig. 6E), and red nucleus (WMD = 0.85, 95% CI = 0.15–1.54, *p* = 0.02, I^2^ = 44%; Fig. 6H). When the article of Wang *et al*.^37^ was removed based on sensitivity analysis, we still observed an increase of iron concentration in the putamen (WMD = 0.82, 95% CI = 0.33–1.30, *p* = 0.001, I^2^ = 0%; Fig. 6F). Significant heterogeneity (I^2^ = 87%, *p* < 0.00001) was detected in the globus pallidus group (Fig. 6B), which was attributed to Han *et al*.^31^ as determined by a sensitivity analysis. Meta-analysis after exclusion of this paper showed a significant increase of iron concentration in the globus pallidus (WMD = 1.76, 95% CI = 0.98–2.54, *p* < 0.0001, I^2^ = 0%; Fig. 6C). The publication biases were acceptable as determined by funnel plots (Fig. 7).

### Structure by structure analyses of results from individual studies and meta-analyses

It is known that inferences can be particularly prone to Type-I error in studies based on a small number of papers, especially with a small sample size^39^. Therefore, we herein elaborated on the results reported in each study combining the results of meta-analyses and the methodological factors that could have contributed to discrepancies in a brain structure-based fashion.

#### Substantia nigra

As expected, an elevation of iron concentration was found in the substantia nigra in all the three types of measurements (Table 2). This was in line with the majority of the 29 articles we analyzed. Except for the three that did not show a change in postmortem samples^10^,^12^,^16^, the other 26 articles reported a trend toward or a statistically significant increase in iron content in the substantia nigra regardless of the type of measurement (postmortem, SWI or R2*). As a note, three postmortem iron analyses^12^,^14^,^16^ indicated that the pars compacta and reticulata were not discriminated during the measurement, while the other six studies did not state the relevant information to make this determination.

#### Putamen

Both postmortem and SWI meta-analyses showed an iron overload in PD patients. However, when individual articles describing postmortem samples were analyzed, we found that only one study reported a significant increase in iron content^9^, while the other five were completely negative with mixed trends^2^,^4^,^11^,^12^,^13^. Although the results of our meta-analysis suggested a significant increase in iron content in the putamen of PD patients in postmortem samples, caution should be taken in the interpretation of these results as one positive study^9^ dominated the other five negative ones in the analysis (Fig. 2G). For SWI, an iron overload was suggested in the putamen based on both random and fixed effects models. Results of two independent studies showed elevated iron content in this structure^31^,^37^, whereas the other five were not significantly different^33^,^34^,^35^,^36^,^38^. One of the positive studies^37^ was removed following a sensitivity analysis, and the remaining one^31^ drove half of the total effect size thereafter in the fixed effects model (Fig. 6F). Taken together, additional studies are needed to confirm iron accumulation in the putamen.

#### Globus pallidus

For SWI, results of six studies suggested a trend toward, or a significant, increase in the level of iron^33^,^34^,^35^,^36^,^37^,^38^, while one showed a decrease in iron content^31^, which was later removed based on a sensitivity analysis. The subsequent meta-analysis returned a significant increase of iron content in the globus pallidus. However, results of either postmortem or R2* meta-analyses did not display significant difference, which was in line with the mixed trends of changes in individual studies.

#### Nucleus caudatus

Similar to globus pallidus, both postmortem and R2* meta-analyses returned no significant difference with mixed trends in iron content in the individual studies. Results of pooled SWI analysis showed a significant increase of iron content in PD patients. There were six studies that showed a significant^31^,^37^ or a trend of increase^33^,^34^,^35^,^36^ in iron levels in the nucleus caudatus while only one study suggested a trend of decrease^34^.

#### Frontal lobe, temporal lobe and cerebellum

Although postmortem results of these structures were available, the pooled sample sizes were small (98, 44 and 58, respectively). All the four studies on frontal lobe^2^,^11^,^12^,^14^ and two on cerebellum^2^,^14^ reported negative results. Although one article reported a significant decrease of iron levels in the temporal lobe^17^, two studies showed no change^11^,^13^. Further studies were needed to clarify iron levels in these structures.

#### Red nucleus

No available studies using postmortem samples fit our criteria. Results of R2* and SWI pooled analyses suggested an increase of iron levels in the red nucleus. For the R2* analyses, four studies reported a significant increase^3^ or an increasing trend^20^,^27^,^29^, whereas one showed a decreasing trend^30^. For the SWI analyses, seven out of eight studies reported no remarkable changes, among which three showed a decreasing trend^32^,^34^,^38^ and four an increasing trend in iron content^32^,^36^,^37^,^38^. In comparison, the study that showed significantly elevated iron content in PD patients^33^ drove roughly half of the total effect size (Fig. 6H). Noteworthy, two PD groups (advanced and mild disease stage) were included in this study that had the same control group^33^. The advanced PD group was chosen for the current analysis to compare with postmortem samples that are usually obtained at late stage PD. When the mild group was included, results of SWI meta-analyses were not affected except in the red nucleus. There was no significant increase of iron content detected (Figs S1 and S2), suggesting that the severity of PD might be a factor affecting iron deposits in the red nucleus. As a note, the mild stage in this study^33^ was Hoehn and Yahr scale <1.5, which appeared milder than normally defined.

#### Thalamus and white matter

No qualified study using postmortem samples was available. Results of both R2* and SWI meta-analyses suggested no association of iron levels with PD in the thalamus and white matter of the brain. Furthermore, all of the selected individual studies^31^,^34^,^35^,^36^,^37^ returned negative results.

## Discussion

Iron dysregulation is frequently associated with neurodegenerative disorders, including Huntington disease, Alzheimer’s disease, amyotrophic lateral sclerosis, and frontotemporal lobar degeneration^40^,^41^. Nonetheless, it remains unclear whether such defect is a cause or a consequence of neurodegeneration. A large body of evidence suggests abnormal iron levels in the brains of PD patients and a role for iron dysregulation in PD pathogenesis^42^,^43^,^44^. Our study represents the first meta-analysis that systematically assesses iron levels in various brain regions of PD patients by postmortem measurements and by MRI (R2* and SWI). Our analysis confirms a perturbed iron homeostasis in the substantia nigra and suggests that an increase in iron levels may also occur in the putamen and red nucleus (Table 2).

Some caveats in regard to the scope of this meta-analysis must be taken into account. First, in the postmortem analyses different iron quantification methods (SPH, AA, COL, ICP and MS) have been used. The differential sensitivity and specificity of these methods may contribute to an elevated heterogeneity. Second, disease stage and age may be two influencing factors when evaluating iron concentration in the brain^40^,^45^,^46^, which unfortunately is not addressed in the current study due to incomplete information and limited sample size. For example, the inclusion of a sub-group of mild-stage PD patients results in a loss of significance in iron levels in the red nucleus of SWI meta-analysis.

It is well recognized that iron overload contributes to oxidative stress through Fenton reaction, promoting the death of dopaminergic neurons in the substantia nigra^47^. Such iron accumulation is known to be associated with increased ferritin and neuromelanin iron loads^48^,^49^, as well as increased expression of divalent metal transporter 1 that may contribute to PD pathogenesis via its capacity of transporting ferrous iron^47^. Furthermore, aggregation of α-synuclein can be accelerated when bound with free iron^50^. However, it remains unclear whether iron deposit triggers or accelerates neurodegeneration, or if they are a secondary event due to neuronal degeneration. Therefore, it is important to determine the timing of iron deposit in substantia nigra during the pathogenesis of PD. Because postmortem measurements are usually made in a very late stage of PD, future longitudinal studies of iron contents are warranted^47^. Consistent results obtained from postmortem, R2*, and SWI measurements suggest that longitudinal evaluation of iron content in the substantia nigra can be appropriately made by MRI methods.

It appears that the MRI methods of R2* and SWI do not completely match the postmortem results, presumably the latter being the standard. Iron deposit is detected by SWI in the globus pallidus and nucleus caudatus, but these are inconsistent with the postmortem observations. Results from R2* studies also suggest an inconsistency in the putamen as both postmortem and SWI effects show an iron overload. Loss of striatal dopamine in PD is most prominent in sub-regions of the putamen^51^, which may be associated with an increase in iron levels. However, this may be a weak argument considering that the postmortem iron increase in this structure is driven by a single study as noted in the Results. It has previously been proposed that SWI is more specific and precise than other methods to estimate brain iron content^52^. Our results suggest that both methods have weakness in measuring iron content. The iron signal determined by R2* may be disrupted by calcification^53^ and lipid content^54^, and the output value is a weighted summation of magnetic properties from both local and surrounding tissues^28^. Intrinsic defects of SWI include a difficulty in distinguishing diamagnetic and paramagnetic susceptibility owning to the convoluting effect of the dipole fields^55^. There are also limitations of MRI *per se*, such that myelin, especially small myelinated fibers, cannot be easily distinguishable from iron deposition^46^, and the phase value of MRI reflects not only non-heme iron deposited in the tissue but also the heme iron in hemosiderin or in circulating blood^56^. Microbleeds may also be a confounding factor especially when brain iron content is estimated in older adults^57^. Given the MRI phase’s nonlocal behavior, one should pay attention to the signal interference of adjacent structures. For example, the red nucleus lies adjacent to substantia nigra in the midbrain and is likely high in iron levels due to its proximity^58^. In other words, the differences detected in iron levels in the red nucleus may arise from the adjacent substantia nigra, instead of from the structure itself. Increased iron levels in red nucleus are associated with levodopa-induced dyskinesia of PD^3^. Future postmortem studies are warranted to confirm iron deposit in this structure. This is also the case for the putamen and globus pallidus, due to their relative proximity. Recently, quantitative susceptibility mapping (QSM), a potentially superior method to measuring iron content *in vivo*, has been applied to measure PD-related iron deposition and progression^28^. By this method, Guan *et al*.^59^ have recently reported a distinct pattern of iron accumulation according to disease stage, with iron spreading from the substantia nigra in early stages to the substantia nigra, red nucleus and globus pallidus in later stages. This could explain the aforementioned discrepancy in the red nucleus when the mild PD group is included, as well as provide a potential explanation for inconsistent findings between neuropathology and MRI techniques.

In conclusion, the current meta-analysis corroborates iron overload in substantia nigra and suggests such iron homeostasis defect in the putamen (by postmortem and SWI, but not R2*) and the red nucleus (by R2* and SWI; no data by postmortem) of PD patients. Both the R2* or SWI techniques may not authentically reflect iron changes in brain regions other than substantia nigra. Our results offer a comprehensive understanding of iron loads in different brain regions in association with PD, and contribute to the evaluation of measuring accuracy of iron concentration by MRI methods.

## Methods

### Literature Search Strategy

Literature related to iron and Parkinson’s disease were searched in four databases including Medline via PubMed, Web of Science, the Cochrane Central Register of Controlled Trials (CENTRAL) and Embase via OVID, dated till 19^th^ November 2015. The keywords for iron and Parkinson’s disease are “iron” or “Fe” and “Parkinson disease”, “Parkinson’s disease”, “Parkinsons disease” or “Parkinsonian”, respectively.

### Study Selection

Based on the keywords, titles and abstracts of the identified publications were screened. Following an exhaustive examination of the literature contents, articles were included according to our selection criteria: population (idiopathic PD patients), comparators (individuals free of neurological disorders), outcome measurement (iron content in brain regions), and language (articles written in English or Chinese). Review articles, qualitative and semi-quantitative studies were excluded.

### Data Extraction

The literature search and data extraction were conducted by two researchers (Qing-Qing Zhuang and Jian-Yong Wang) independently. In the case of a dispute, a third investigator was included to discuss and reach an agreement. The following data was extracted: sample size, age, sex, PD diagnosis, iron detection methods, the type of samples, clinical scores, and iron content or R2* value or phase value in brain regions. Assessment of the detailed information was listed in Table 1. As shown in this table, the disease severity (Hoehn and Yahr scale) was not provided by all the included studies and the provided else information was also varied in forms including UPDRS score, UPDRS motor score, and/or disease duration. Therefore, we did not include the disease severity as a source of variance in the analysis.

Iron quantification methods employed in the postmortem study of brain samples included spectrophotometry (SPH), atomic absorption (AA), colorimetry (COL), inductively coupled plasma spectroscopy (ICP) and Mössbauer spectroscopy (MS). To be consistent in brain weights, a conversion of dry weight to wet weight was applied based on a dry/wet ratio as suggested in previous studies^60^,^61^. The SWI signal phase is orientation-dependent and nonlocal^55^. As a result, the phase value appears to be either positively or negatively correlated with iron concentration depending on the orientation relative to the Bo field^62^. Thus, a conversion from SWI phase value to iron concentration was applied based on formulas suggested in previous studies^35^,^36^; that is, concentration = 397.72 × (phase value) + 3.4097 (extracted from Fig. 1 of ref. 36) for the studies of positive setting^31^,^34^,^36^, and concentration = −128.23 × (phase value) + 3.1897 (extracted from Fig. 2 of ref. 35) for the studies of negative setting^32^,^33^,^35^,^37^,^38^.

### Quality Assessment

The Newcastle-Ottawa Scale^63^ was employed to assess the quality of the chosen studies. This tool classified studies in three broad perspectives: selection of the study groups, comparability of the groups, and ascertainment of either exposure or outcome of interest for the studies. Semi-quantitative measurement using a star system assesses the quality of study. The highest quality studies can get a maximum of nine stars.

### Statistical Analysis

Eleven postmortem analysis and 22 MRI analysis articles were eventually selected for our meta-analysis. Means, standard deviations (or standard errors), and the number of samples were extracted in each study. Meta-analyses were conducted within the studies of the same brain region after sorting into their respective quantitative groups of postmortem analysis, R2* and SWI. In the case that the same data appeared in multiple studies, the data were used only once. All of the analyses were performed using Review Manager 5.2 for Windows (http://ims.cochrane.org/recman). A two-tailed *p* value <0.05 was considered statistically significant. Weighted mean difference (WMD) was regarded as an effect size. Q-statistics and I^2^ were used for assessing the heterogeneity^64^,^65^. A random effects model was applied when heterogeneity was found by Q-statistics or when I^2^ > 50%. A fixed effects model was applied otherwise.

## Additional Information

**How to cite this article**: Wang, J.-Y. *et al*. Meta-analysis of brain iron levels of Parkinson’s disease patients determined by postmortem and MRI measurements. *Sci. Rep*. **6**, 36669; doi: 10.1038/srep36669 (2016).

**Publisher’s note:** Springer Nature remains neutral with regard to jurisdictional claims in published maps and institutional affiliations.

## Supplementary Material

Supplementary Information

## Figures and Tables

**Table 1:** Characteristics of the 33 studies included for meta-analyses.

Article	Healthy controls			PD patients			PD diagnosis	Detection	Method type	UPDRS score	UPDRS motor score	H-Y scale	Disease duration	Publication Quality Assessment
	n	Age^a^	Gender (F/M)	n	Age^a^	Gender (F/M)								
Yu *et al*.^17^	10	84.6 ± 1.5	6/4	10	82.7 ± 1.7	4/6	UK PD Brain Bank criteria	ICP	Postmortem	—	—	—	—	******
Loeffler *et al*.^12^	8	74.6 ± 7.6	4/4	14	74.9 ± 8.7	5/9	Pathological examination	COL	Postmortem	—	—	—	—	****
Griffiths *et al*.^11^	6	83.3 ± 2.1	—	6	83.6 ± 2.4	—	Pathological examination	AA	Postmortem	—	—	—	—	******
Dexter *et al*.^2^	34	81.3 ± 1.5	21/13	27	74.9 ± 1.4	11/16	Clinical and pathological examination	ICP	Postmortem	—	—	—	—	*****
Riederer *et al*.^4^	4	73 (68–78)	3/1	13	76 (68–82)	7/6	Pathological examination	SPH	Postmortem	—	—	—	—	*****
Sofic *et al*.^13^	8	75.3 (66–86)	4/4	8	71.3 ± 12.5	4/4	Pathological examination	SPH	Postmortem	—	—	—	7.5 ± 3.4	******
Visanji *et al*.^15^	3	62.7 (47–78)	1/2	3	69.3 (56–79)	1/2	Pathological examination	AA	Postmortem	—	—	—	21 ± 3.8	*****
Wypijewska *et al*.^16^	29	61–85	—	17	61–85	—	Clinical and pathological examination	MS	Postmortem	—	—	—	—	******
Galazka-Friedm *et al*.^10^	8	64 ± 6	—	6	70 ± 4	—	Clinical and pathological examination	MS	Postmortem	—	—	4–5	4–7	*****
Uitti *et al*.^14^	12	70	4/8	9	73	3/6	Pathological examination	AA	Postmortem	—	—	—	—	****
Chen *et al*.^9^	6	—	—	10	—	—	—	AA	Postmortem	—	—	—	—	****
Gorell *et al*.^18^	10	60.0 ± 8.7	5/5	13	65.2 ± 12.7	2/11	Clinical diagnosis	3T	R2*	—	—	1.5–3.0	3–13	*****
Graham *et al*.^19^	25	64.0 ± 6.6	6/7	21	61.4 ± 7.3	10/11	UK PD Brain Bank criteria	1.5T	R2*	—	—	—	11.1 ± 4.5	******
Martin *et al*.^20^,^b^	11	55.9 ± 7.3	4/7	22	61.9 ± 9.0	8/14	Published criteria^66^	3T	R2*	—	16.7 ± 7.1	—	3.2 ± 1.7	*******
				19	60.3 ± 8.4	6/13				—	16.9 ± 7.5	—	2.9 ± 1.6	
Du *et al*.^21^	29	59.6 ± 6.7	17/12	40	60.7 ± 8.3	17/23	Published criteria^66^	3T	R2*	—	23.4 ± 15.2	1.8 ± 0.6	4.2 ± 4.7	******
Bunzeck *et al*.^22^	20	66.0 ± 9.1	10/10	20	66.3 ± 9.0	9/11	Queens Square Brain Bank criteria^67^	3T	R2*	34.6 ± 17.4	—	—	—	******
Lee *et al*.^23^	21	60.0 ± 6.1	9/12	29	59.1 ± 7.6	12/17	UK PD Brain Bank criteria	3T	R2*	—	25.5 ± 9.2	2.05 ± 0.5	2.5 ± 1.9	*******
Lewis *et al*.^3^	23	59.9 ± 7.0	17/12	38	60.6 ± 8.0	17/23	Published criteria^66^	3T	R2*	—	23.8 ± 15.4	1.8 ± 0.6	4.4 ± 4.7	*****
Rossi *et al*.^24^	21	66 (58–80)	17/4	37	69 (42–86)	18/19	Clinical diagnosis	3T	R2*	—	—	—	—	*****
Ulla *et al*.^25^	26	57.0 ± 8.5	17/9	27	60.2 ± 10.7	14/13	PD Society Brain Bank^68^	1.5T	R2*	—	12.1 ± 8.5	1.9 ± 0.7	5.7 ± 4.4	******
Rossi *et al*.^26^	19	65 (58–80)	15/4	25	73 (44–87)	14/11	Clinical diagnosis	3T	R2*	—	—	—	—	*****
Barbosa *et al*.^27^	30	64 ± 7	21/9	20	66 ± 8	8/12	UK PD Brain Bank criteria	3T	R2*	—	—	2.3 ± 0.6	8.1 ± 4.2	******
Murakami *et al*.^30^	21	69.7 ± 8.6	12/9	21	72.0 ± 7.5	12/9	UK PD Brain Bank criteria	3T	R2*	—	—	2 (1–3)	2.7 ± 2.3	*****
He *et al*.^29^	35	60.5 ± 6.5	14/21	44	58.0 ± 8.8	19/25	UK PD Brain Bank criteria	3T	R2*	—	15.6 ± 6.2	1.4 ± 0.5	2.8 ± 1.6	****
Du *et al*.^28^	47	62.2 ± 8.8	24/23	47	65.8 ± 10.1	25/22	UK PD Brain Bank criteria	3T	R2*	39.6 ± 24.8	21.8 + 15.2	—	5.5 ± 4.8	*****
Zhang *et al*.^34^	26	57.3 ± 11.6	12/14	40	58.7 ± 12.8	19/21	UK PD Brain Bank criteria	3T	SWI	—	19.0 ± 7.8	—	3.6 ± 2.9−	******
Jin *et al*.^35^	45	55.4 ± 14.9	19/26	45	56.3 ± 10.9	14/31	UK PD Brain Bank criteria	3T	SWI	15.1 ± 9.3	12.0 ± 7.1	—	—	******
Wang *et al*.^32^	14	64.3 ± 12.7	7/7	20	67.2 ± 10.7	10/10	Clinical diagnosis	3T	SWI	—	—	—	2.8 ± 2.8	******
Wang *et al*.^37^	44	59.4 ± 11.8	23/21	16	63.3 ± 10.6	7/9	UK PD Brain Bank criteria	1.5T	SWI	—	—	—	2.5 ± 1.7	*****
Han *et al*.^31^	20	55.9 ± 6.2	8/12	15	57.4 ± 7.1	8/7	UK PD Brain Bank criteria	3T	SWI	23.0 ± 5.6	—	2.2 ± 0.5	2.5 ± 1.6	*****
Kim *et al*.^36^	25	56.2 ± 6.5	13/12	30	57.6 ± 6.8	11/19	UK PD Brain Bank criteria	3T	SWI	—	24.5 ± 8.4	1.7 ± 0.5	1.7 ± 1.1	*****
Wu, *et al*.^33^	40	66.5 ± 6.0	18/22	54	65.6 ± 5.8	21/33	UK PD Brain Bank criteria	3T	SWI	—	—	≥1.5	—	****
Huang, *et al*.^38^	19	65.0 ± 9.0	—	30	68.0 ± 9.0	6/24	—	3T	SWI	—	—	—	—	****

^a^Data in this column are presented as mean ± SD or Range or Median (Range) or Mean (Range) or the detail ages; ^b^In this study the patient group with n = 22 is for mid-brain images including substantia nigra and red nucleus, and the one with n = 19 is for forebrain images including globus pallidus, putamen, nucleus caudatus, and white matter. UPDRS, Unified Parkinson’s Disease Rating Scale; H-Y, Hoehn and Yahr; ICP, inductively coupled plasma spectroscopy; COL, colorimetry; AA, atomic absorption; SPH, spectrophotometry; MS, Mössbauer spectroscopy.

**Table 2:** A summary of changes in brain iron levels of PD patients based on the current meta-analysis.

	Postmortem			R2*			SWI		
	Change	*p*	n	Change	*p*	n	Change	*p*	n
**Substantia nigra**	↑	0.01	173	↑	<10^−5^	631	↑	<10^−4^	465
	↑^a^	0.004	161	↑^a^	<10^−5^	556			
**Putamen**	↑	0.002	100	—	0.74	446	↑	0.04	431
							↑^a^	0.001	371
**Globus pallidus**	—	0.72	104	—	0.37	500	—	0.20	431
							↑^a^	<10^−4^	396
**Nucleus caudatus**	—	0.47	117	—	0.80	437	↑	0.0003	431
**Frontal lobe**	—	0.33	98	NA	NA	NA	NA	NA	NA
**Temporal lobe**	—	0.11	44	NA	NA	NA	NA	NA	NA
**Cerebellum**	—	0.14	58	NA	NA	NA	NA	NA	NA
**Red nucleus**	NA	NA	NA	↑	0.002	265	↑	0.02	465
**Thalamus**	NA	NA	NA	—	0.81	182	—	0.94	306
**White matter**	NA	NA	NA	—	0.07	117	—	0.93	211

^↑^Increased iron level in PD.

^—^No change of iron level in PD.

NA, no data available.

^a^Analyzed after heterogeneity was removed.
