# Supplementary material for: Meta-analysis of brain iron levels of Parkinson’s disease patients determined by postmortem and MRI measurements
Source: Sci Rep. 2016 Nov 9;6:36669. doi: 10.1038/srep36669 (PMC5101491; doi:10.1038/srep36669)
Supplement: Supplementary Information [file srep36669-s1.pdf]

Meta-analysis of brain iron levels of Parkinson's disease patients determined by postmortem and MRI measurements

Jian-Yong Wang[a,b,*], Qing-Qing Zhuang[a,*], Lan-Bing Zhu[a], Hui Zhu[a], Ting Li[a], Rui Li[b], Song-Fang Chen[a], Chen-Ping Huang[b,#], Xiong Zhang[a,#], Jian-Hong Zhu[b,c,#]

Supplementary Table S1. The disease comorbidity and diagnostic performance of the included 33 studies

Article	PD patients	Controls
Yu et al., 2013[17]	NM	No neurologic or psychiatric disease
Loeffler et al., 1995[12]	NM	Other than CNS disease and lacking any form of pathology at autopsy
Griffiths et al., 1993[11]	NM	No neurologic or psychiatric disease
Dexter et al., 1989[2]	NM	No neurologic or psychiatric disease
Riederer et al., 1989[4]	NM	Heart failure (2), lung embolism (1), and labile hypertonia (1)
Sofic et al., 1988[13]	Bronchopneumonia (6), pulmonary embolism after leg vein thrombosis and hypertensive heart disease (1), and cardiac arrest after colon carcinoma (1)	Died without any evidence of neurological or psychiatric disease. Bronchopneumonia (2), myocardial infarction (1), pulmonary thromboembolism and arteriosclerotic cerebrovascular disease (1), coronary arteriosclerosis and old infarction (1), coronary thrombosis (1), corpulmonale, chronic bronchitis and emphysema (1), and pleural mesothelioma (1)
Visanji et al., 2013[15]	NM	NM
Wypijewska et al., 2010[16]	Died at advanced stages of the disease	No neurological diseases (myocardial infarct in most cases)
Galazka-Friedm et al., 1996[10]	Unrelated to the neurological disease (pneumonia, heart disease).	Died without any symptoms of an extrapyramidal disease
Uitti et al., 1989[14]	NM	NM
Chen et al., 1993[9]	NM	NM
Gorell et al., 1995[18]	Absence of either neuroleptic drug intake or a CNS disorder with Parkinson-like and other neurologic deficits (e.g. cerebellar, corticospinal, amyotrophic, autonomic, or supranuclearophthalmoplegic signs)	Free of CNS disease
Graham et al., 2000[19]	NM	Volunteer or spouses of patients with

		Parkinson’s disease
Martin et al., 2008[20]	NM	Free of neurologic or psychiatric disease
Du et al., 2012[21]	NM	NM
Bunzeck et al., 2013[22]	No psychiatric or neurological disorders other than PD	No history of neurological, psychiatric or medical disorders or any current medical problems
Lee et al., 2013[23]	No microvascular lesions found on brain MRI	No neurologic or psychiatric disease
Lewis et al., 2013[3]	Free of major acute medical issues such as liver, kidney or thyroid abnormalities, or deficiencies of B12 or folate.	NM
Rossi et al., 2013[24]	Exclusion criteria: Alzheimer's disease or any other type of dementia that was diagnosed within 1 year preceding the study; alcohol or drug addiction; pregnancy, severe general illnesses such as cardiac, lung, or gastrointestinal disease; liver or kidney malfunction; active malignant neoplasm; and neurological or psychiatric disease.	Hospital employees and the patients' spouses: the inclusion criterion was a clinical examination with no abnormal findings. The exclusion criterion was a history of cerebrovascular attack.
Ulla et al., 2013[25]	NM	No neurologic or psychiatric disease
Rossi et al., 2014[26]	Exclusion criteria: presence of Alzheimer’s disease or other dementia; other severe disease such as heart, lung, or gastrointestinal disease; hypofunction of liver or kidney; active cancer; neurological or psychiatric disease; a history of cerebrovascular attack; contraindications for MRI; alcohol or drug addiction; and pregnancy.	A clinical neurological examination with no abnormal findings
Barbosa et al., 2015[27]	No history of other neurological and psychiatric disorders	No history of neurological disease, hypertension or diabetes
Murakami et al., 2015[30]	NM	No history of neurologic or psychiatric diseases
He et al., 2015[29]	No presence of dementia; no other neurological or psychiatric disorders	NM
Du et al., 2016[28]	No history of neurological or psychiatric disease other than PD	No neurologic or psychiatric disease
Zhang et al., 2010[34]	Without dementia, no history of neurologic or psychiatric disease and neurologic sequelae induced by brain trauma	Hospital staff and local community
Jin et al., 2011[35]	Exclusion criteria: signs of upper and/or lower motor neuron dysfunction; orthostatic hypotension within three years of PD onset; cognitive impairment as assessed by the Mini-Mental State Examination; hepatic and/ or renal dysfunction.	No history of neurologic/psychiatric disorders and no abnormal signal in structural magnetic resonance imaging.

Wang et al., 2011[32]	NM	No abnormal signal in MRI examination; no history of central nervous system disease; no drug abuse, smoking and alcohol history
Wang et al., 2012[37]	NM	NM
Han et al., 2013[31]	NM	No neurologic or psychiatric disease
Kim et al., 2014[36]	NM	NM
Wu, et al., 2014[33]	NM	NM
Huang, et al., 2010[38]	NM	Healthy volunteers

NM, not mentioned. The numbers in parenthesis indicate 'n' for each comorbidity.

(A) Nucleus caudatus

Study or Subgroup	Parkinson's disease Mean	SD	Total	Control Mean	SD	Total	Weight	Mean Difference IV, Fixed, 95% CI
Han et al., 2013	14.11	1.75	15	13.67	0.87	20	18.0%	0.44 [-0.52, 1.40]
Huang et al., 2010	11.14	1.54	30	10.76	1.67	19	19.3%	0.38 [-0.55, 1.31]
Jin et al., 2011	15.63	4.23	45	14.99	2.18	45	8.7%	0.64 [-0.75, 2.03]
Kim et al., 2014	16.53	5.17	30	15.74	5.17	25	2.2%	0.79 [-1.95, 3.53]
Wang et al., 2012	18.84	4.33	16	18.43	3.72	44	2.9%	0.41 [-1.98, 2.80]
Wu et al., 2014	12.12	1.83	54	11.01	1.15	40	45.9%	1.11 [0.51, 1.71]
Zhang et al., 2010	18.14	4.79	40	19.25	4.93	26	2.9%	-1.11 [-3.52, 1.30]
**Total (95% CI)**			**230**			**219**	**100.0%**	**0.72 [0.31, 1.12]**

Heterogeneity: $Chi^2 = 4.74$, df = 6 (P = 0.58); $I^2 = 0\%$
Test for overall effect: Z = 3.42 (P = 0.0006)

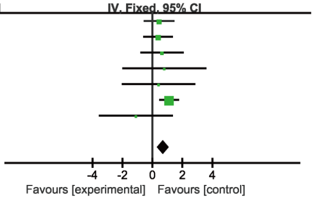

(B) Globus pallidus

Study or Subgroup	Parkinson's disease Mean	SD	Total	Control Mean	SD	Total	Weight	Mean Difference IV, Random, 95% CI
Han et al., 2013	14.73	0.87	15	16.04	0.65	20	20.3%	-1.31 [-1.83, -0.79]
Huang et al., 2010	13.83	3.08	30	11.78	1.8	19	18.1%	2.05 [0.68, 3.42]
Jin et al., 2011	17.17	4.62	45	16.4	2.95	45	17.3%	0.77 [-0.83, 2.37]
Kim et al., 2014	17.73	5.57	30	17.33	5.97	25	12.1%	0.40 [-2.67, 3.47]
Wang et al., 2012	18.51	6.63	16	14.86	2.7	44	11.2%	3.65 [0.30, 7.00]
Wu et al., 2014	14.64	3.48	54	12.94	1.8	40	19.0%	1.70 [0.62, 2.78]
Zhang et al., 2010	39.75	21.5	40	38.59	23.73	26	1.9%	1.16 [-10.14, 12.46]
**Total (95% CI)**			**230**			**219**	**100.0%**	**1.04 [-0.61, 2.69]**

Heterogeneity: $Tau^2 = 3.41$; $Chi^2 = 46.02$, df = 6 (P < 0.00001); $I^2 = 87\%$
Test for overall effect: Z = 1.24 (P = 0.22)

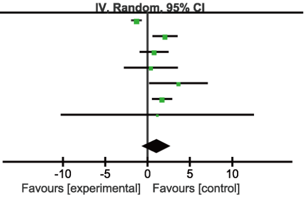

(C) Globus pallidus*

Study or Subgroup	Parkinson's disease Mean	SD	Total	Control Mean	SD	Total	Weight	Mean Difference IV, Fixed, 95% CI
Huang et al., 2010	13.83	3.08	30	11.78	1.8	19	27.0%	2.05 [0.68, 3.42]
Jin et al., 2011	17.17	4.62	45	16.4	2.95	45	19.7%	0.77 [-0.83, 2.37]
Kim et al., 2014	17.73	5.57	30	17.33	5.97	25	5.3%	0.40 [-2.67, 3.47]
Wang et al., 2012	18.51	6.63	16	14.86	2.7	44	4.5%	3.65 [0.30, 7.00]
Wu et al., 2014	14.64	3.48	54	12.94	1.8	40	43.1%	1.70 [0.62, 2.78]
Zhang et al., 2010	39.75	21.5	40	38.59	23.73	26	0.4%	1.16 [-10.14, 12.46]
**Total (95% CI)**			**215**			**199**	**100.0%**	**1.63 [0.92, 2.34]**

Heterogeneity: $Chi^2 = 3.51$, df = 5 (P = 0.62); $I^2 = 0\%$
Test for overall effect: Z = 4.49 (P < 0.00001)

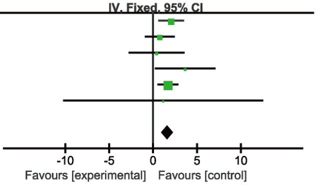

(D) Substantia nigra

Study or Subgroup	Parkinson's disease Mean	SD	Total	Control Mean	SD	Total	Weight	Mean Difference IV, Random, 95% CI
Han et al., 2013	34.38	2.19	15	22.54	1.76	20	15.8%	11.84 [10.49, 13.19]
Huang et al., 2010	18.71	3.33	30	16.27	3.21	19	15.4%	2.44 [0.57, 4.31]
Jin et al., 2011	27.04	4.23	45	23.58	3.59	45	15.6%	3.46 [1.84, 5.08]
Kim et al., 2014	23.3	9.15	30	17.33	7.95	25	12.3%	5.97 [1.45, 10.49]
Wang et al., 2011	69.81	22.7	20	50.63	21.8	14	3.5%	19.18 [4.03, 34.33]
Wang et al., 2012	32.39	4.87	16	24.48	7.1	44	14.0%	7.91 [4.73, 11.09]
Wu et al., 2014	19.73	1.78	54	16.14	2.44	40	16.1%	3.59 [2.70, 4.48]
Zhang et al., 2010	59.92	20.34	40	51.96	16.52	26	7.2%	7.96 [-0.99, 16.91]
**Total (95% CI)**			**250**			**233**	**100.0%**	**6.46 [3.24, 9.69]**

Heterogeneity: $Tau^2 = 16.66$; $Chi^2 = 124.23$, df = 7 (P < 0.00001); $I^2 = 94\%$
Test for overall effect: Z = 3.93 (P < 0.0001)

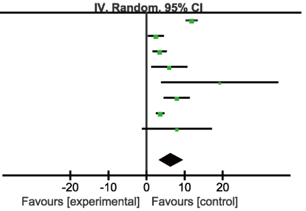

(E) Putamen

Study or Subgroup	Parkinson's disease Mean	SD	Total	Control Mean	SD	Total	Weight	Mean Difference IV, Random, 95% CI
Han et al., 2013	12.94	1.09	15	11.84	0.65	20	25.6%	1.10 [0.48, 1.72]
Huang et al., 2010	11.27	2.69	30	11.52	2.56	19	16.9%	-0.25 [-1.75, 1.25]
Jin et al., 2011	12.29	4.87	45	12.29	4.1	45	13.9%	0.00 [-1.86, 1.86]
Kim et al., 2014	10.57	4.77	30	8.98	3.18	25	12.0%	1.59 [-0.52, 3.70]
Wang et al., 2012	17.3	5.07	16	11.83	4.05	44	8.6%	5.47 [2.71, 8.23]
Wu et al., 2014	12.72	2.59	54	12.29	2.69	40	21.0%	0.43 [-0.65, 1.51]
Zhang et al., 2010	19	13.26	40	18.28	13.44	26	2.0%	0.72 [-5.88, 7.32]
**Total (95% CI)**			**230**			**219**	**100.0%**	**1.00 [0.04, 1.97]**

Heterogeneity: $Tau^2 = 0.84$; $Chi^2 = 15.23$, df = 6 (P = 0.02); $I^2 = 61\%$
Test for overall effect: Z = 2.04 (P = 0.04)

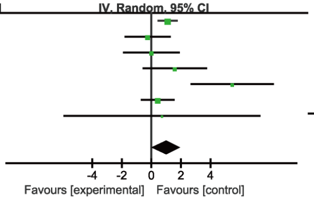

(F) Putamen*

Study or Subgroup	Parkinson's disease Mean	SD	Total	Control Mean	SD	Total	Weight	Mean Difference IV, Fixed, 95% CI
Han et al., 2013	12.94	1.09	15	11.84	0.65	20	58.6%	1.10 [0.48, 1.72]
Huang et al., 2010	11.27	2.69	30	11.52	2.56	19	10.0%	-0.25 [-1.75, 1.25]
Jin et al., 2011	12.29	4.87	45	12.29	4.1	45	6.5%	0.00 [-1.86, 1.86]
Kim et al., 2014	10.57	4.77	30	8.98	3.18	25	5.1%	1.59 [-0.52, 3.70]
Wu et al., 2014	12.72	2.59	54	12.29	2.69	40	19.3%	0.43 [-0.65, 1.51]
Zhang et al., 2010	19	13.26	40	18.28	13.44	26	0.5%	0.72 [-5.88, 7.32]
**Total (95% CI)**			**214**			**175**	**100.0%**	**0.79 [0.31, 1.26]**

Heterogeneity: $Chi^2 = 4.47$, df = 5 (P = 0.48); $I^2 = 0\%$
Test for overall effect: Z = 3.24 (P = 0.001)

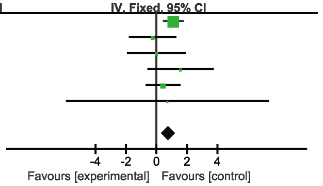

(G) Thalamus

Study or Subgroup	Parkinson's disease Mean	SD	Total	Control Mean	SD	Total	Weight	Mean Difference IV, Fixed, 95% CI
Han et al., 2013	6.65	0.18	15	6.65	0.6	20	67.5%	0.00 [-0.28, 0.28]
Jin et al., 2011	4.47	1.15	45	4.6	1.54	45	16.6%	-0.13 [-0.69, 0.43]
Kim et al., 2014	3.8	1.59	30	3.41	1.59	25	7.3%	0.39 [-0.45, 1.23]
Wang et al., 2012	4.01	1.69	16	3.88	1.96	44	5.1%	0.13 [-0.88, 1.14]
Zhang et al., 2010	8.51	1.95	40	9.18	2.79	26	3.5%	-0.67 [-1.90, 0.56]
**Total (95% CI)**			**146**			**160**	**100.0%**	**-0.01 [-0.24, 0.22]**

Heterogeneity: $Chi^2 = 2.22$, df = 4 (P = 0.70); $I^2 = 0\%$
Test for overall effect: Z = 0.08 (P = 0.94)

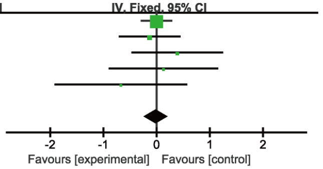

(H) Red nucleus

Study or Subgroup	Parkinson's disease Mean	SD	Total	Control Mean	SD	Total	Weight	Mean Difference IV, Fixed, 95% CI
Han et al., 2013	20.32	3.97	15	19.44	1.54	20	10.6%	0.88 [-1.24, 3.00]
Huang et al., 2010	14.22	3.46	30	14.6	2.18	19	19.0%	-0.38 [-1.96, 1.20]
Jin et al., 2011	20.63	6.03	45	19.22	5.26	45	8.7%	1.41 [-0.93, 3.75]
Kim et al., 2014	27.67	9.15	30	24.1	10.34	25	1.7%	3.57 [-1.64, 8.78]
Wang et al., 2011	21.14	3.38	20	22.42	2.56	14	11.9%	-1.28 [-3.28, 0.72]
Wang et al., 2012	15.34	7.44	16	13.19	5.75	44	2.9%	2.15 [-1.87, 6.17]
Wu et al., 2014	16.14	2.54	54	14.99	2.56	40	43.6%	1.15 [0.11, 2.19]
Zhang et al., 2010	33.28	12.1	40	35.27	9.94	26	1.7%	-1.99 [-7.34, 3.36]
**Total (95% CI)**			**250**			**233**	**100.0%**	**0.58 [-0.10, 1.27]**

Heterogeneity: $Chi^2 = 9.19$, df = 7 (P = 0.24); $I^2 = 24\%$
Test for overall effect: Z = 1.66 (P = 0.10)

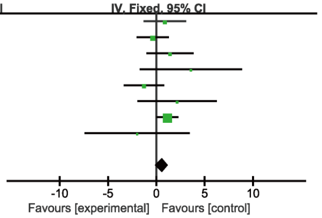

(I) White matter

Study or Subgroup	Parkinson's disease Mean	SD	Total	Control Mean	SD	Total	Weight	Mean Difference IV, Fixed, 95% CI
Jin et al., 2011	2.42	1.41	45	2.55	1.28	45	4.2%	-0.13 [-0.69, 0.43]
Kim et al., 2014	6.33	0.22	30	6.33	0.22	25	94.6%	0.00 [-0.12, 0.12]
Zhang et al., 2010	4.27	2.19	40	4.26	1.93	26	1.3%	0.01 [-1.00, 1.02]
**Total (95% CI)**			**115**			**96**	**100.0%**	**-0.01 [-0.12, 0.11]**

Heterogeneity: $Chi^2 = 0.20$, df = 2 (P = 0.90); $I^2 = 0\%$
Test for overall effect: Z = 0.09 (P = 0.93)

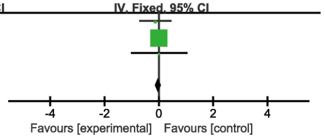

Figure S1. Statistical summaries and forest plots of studies comparing iron concentrations between PD patients and controls by SWI relaxometry. B, D and E, pooled using random-effects models. The others were pooled using fixed-effects models. *, analyzed after heterogeneity was removed.

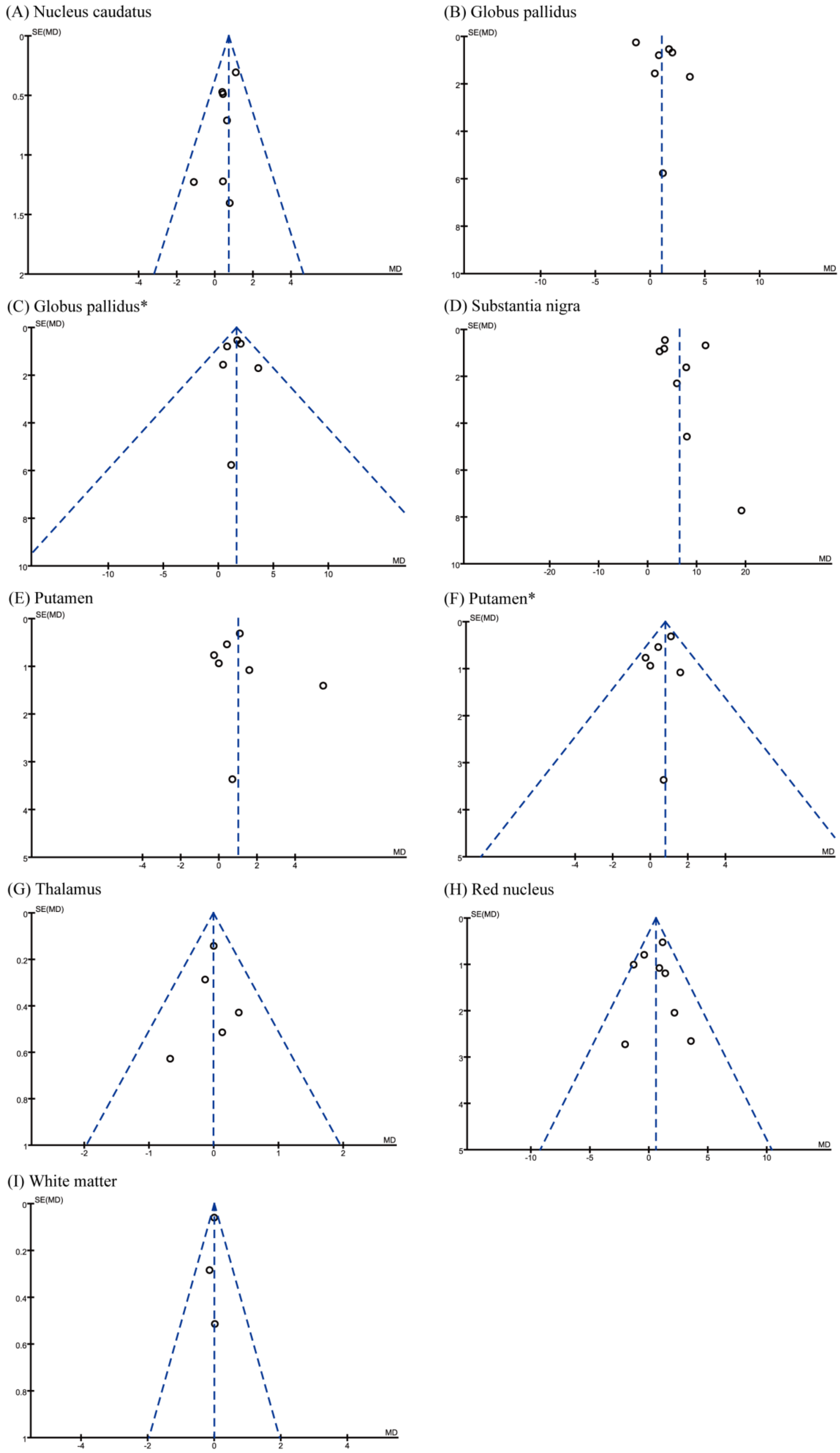

Figure S2. Funnel plots that examine possible publication bias in the studies by SWI. *, analyzed after heterogeneity was removed.
